# Genetic determinants of response to vitamin D supplementation

**DOI:** 10.3389/fnut.2026.1880174

**Published:** 2026-07-15

**Authors:** Nerea Alonso, Inez Schoenmakers, Thomas Kuenzer, Ariane Willems, Huilin Jin, Terry J. Aspray, Stuart H. Ralston

**Affiliations:** 1 Institute of Genetics and Cancer, University of Edinburgh, Edinburgh, United Kingdom; 2 Clinical Institute of Medical and Chemical Laboratory Diagnostics (CIMCL), Medical University of Graz, Graz, Austria; 3 Department of Medicine, Faculty of Medicine and Health Sciences, Norwich Medical School, University of East Anglia, Norwich, United Kingdom; 4 Institute for Medical Informatics, Statistics and Documentation, Medical University of Graz, Graz, Austria; 5 NIHR Newcastle Biomedical Research Centre, Campus for Ageing and Vitality, Newcastle upon Tyne, United Kingdom; 6 Institute for Cellular Medicine, Newcastle University, Newcastle upon Tyne, United Kingdom

**Keywords:** *GC* gene, genetic analysis, nutrition, vitamin D insufficiency, vitamin D supplementation

## Abstract

**Background:**

Genetic variants at the vitamin D binding protein (*GC*), cytochrome P450 family 2 subfamily R member 1 (*CYP2R1*), and 7-dehydrocholesterol reductase (*DHCR7*) loci have been associated with serum 25-hydroxy vitamin D (25(OH)D) concentration, but their role in interpreting the response to vitamin D supplementation is unclear. Here, we investigated associations between common variants at these loci and plasma 25(OH)D concentrations in a randomised dose-ranging trial of vitamin D supplementation in healthy older people.

**Methods:**

The study involved 323 participants of the Vitamin D supplementation in Older People (VDOP) trial, randomly assigned to either 12,000, 24,000, or 48,000 IU colecalciferol monthly for 12 months. Plasma 25(OH)D concentrations were measured by liquid chromatography-mass spectrometry. Variants rs7041 and rs2282679 (*GC*), rs10741657 (*CYP2R1*), and rs12785878 (*DHCR7*) were genotyped by Sanger sequencing. Associations between genotype and 25(OH)D at baseline and post-supplementation were assessed by linear regression analysis with baseline 25(OH)D as a covariate and dose as fixed factor for post-supplementation data.

**Results:**

At baseline, plasma 25(OH)D concentration was associated with the minor allele rs7041-A (*GC*). Post-supplementation, an allele-dose effect was observed with two variants at the *GC* locus. Common homozygotes for rs2282679 and rs7041 had higher 25(OH)D concentrations at all supplement dosages, with lower frequency of 25(OH)D concentrations <50 nmol/L compared to other genotypes (OR(95%CI) = 2.97(1.56–5.65) and 2.84(1.28–6.32), respectively).

**Conclusion:**

Common genetic variants at the *GC* locus influence plasma 25(OH)D concentrations and the response to vitamin D supplementation. Genotyping of *GC* may aid interpreting 25(OH)D concentrations in people who are undergoing supplementation.

## Introduction

Colecalciferol or vitamin D_3_ is a fat soluble vitamin that can be obtained from the diet or generated in the skin from 7-dehydrocholesterol (7-DHC) on exposure to ultraviolet light (1). It enters the bloodstream where about 80% is bound to vitamin D binding protein (VDBP), encoded by the *GC* gene. The remaining 20% is bound to serum albumin and lipoproteins (2) with a very small fraction circulating in its free form. On passage through the liver, cholecalciferol is hydroxylated by *CYP2R1* into 25-hydroxy-vitamin D (25(OH)D), and in the kidneys by *CYP27B1*, into the bioactive form 1,25-dihydroxy-vitamin D (1,25(OH)_2_D). This acts on the gut to increase calcium and phosphate absorption and on bone to regulate bone metabolism.

Vitamin D status is assessed by measurement of plasma concentrations of 25(OH)D (3, 4). There is ongoing debate and differences between guidelines what thresholds of plasma concentration of 25(OH)D are used to define vitamin D deficiency and insufficiency (5, 6). Here, we used 3 commonly thresholds/categories, consistent with United Kingdom guidelines (6): below 25 nmol/L, 25 to 50 nmol/L, and concentrations above 50 nmol/L.

The latest population and patient guidelines from The Endocrine Society (7), the scientific advisory committee of nutrition (SACN), and The European Food Safety Authority (EFSA) recommends against the routine screening of vitamin D for individuals aged between 19 to 74 years without risk factors or symptoms of vitamin D deficiency.

To date, genetic factors have not been considered as risk factors for the management of individuals who continue to have low vitamin D concentrations despite supplementation. However, circulating levels of 25(OH)D has been reported to be influenced by gene variants in *GC* and SNPs in the vicinity of *DHCR7*, the gene coding for 7-DHC, and *CYP2R1.*

The Genome-Wide Association Study (GWAS) by the SUNLIGHT Consortium which included 15 observational studies with over 33,000 individuals (8) identified three loci in the *GC* that were associated with 25(OH)D concentrations. The association was strongest with rs2282679, a noncoding variant at the *GC* gene locus, which is a proxy for rs4588, a variant that encodes a threonine to lysine change at position 436 (T436K) in VDBP. Variants in rs7041 at the *GC* locus were significantly associated with circulating levels of 25(OH)D. This SNP causes a change from aspartic acid to glutamic acid at position 432 (D432Q) of the VDBP. Both non-synonymous rs7041 and rs4588 polymorphisms in *GC* regulate the synthesis of VDBP and are associated with lower 25(OH)D concentrations (9), and may influence the hydroxylation rate of 25(OH)D (25(OH)D half-life) (10).

There are three common VDBP isoforms (Gc1f, Gc1s, Gc2), and over 120 rare variants, which cluster by racial descent and geographical region (11). It had been suggested that low total 25(OH)D concentrations are associated with VDBP genotype, but that this is not reflected in low tissue bioavailability as determined by a relatively high free fraction of 25(OH)D (12). However, evidence is conflicting and inconsistent; other studies showed that total and free 25(OH)D concentrations are highly correlated in studies that either directly measured or calculated free 25(OH)D, and no differences in the free fraction between VDBP genotypes have been found (13, 14), including after vitamin D supplementation (14).

The SUNLIGHT Consortium GWAS also identified associations between 25(OH)D and variants in the rs12785878 SNP, close to *DHCR7* gene. This encodes the enzyme 7-dehydrocholesterol reductase, which converts 7-dehydroxycholesterol (7-DHC) into cholesterol, reducing the availability of 7-DHC as substrate for the generation of pre-vitamin D in the skin by ultraviolet light, the precursor and major source for vitamin D in humans (15).

Further, GWAS identified the rs10741657 SNP, located upstream of the *CYP2R1* gene, which is responsible for 25-hydroxylation of colecalciferol in the liver. Since the SUNLIGHT Consortium GWAS, these findings were validated in subsequent GWAS (16–18).

While multiple common and rare genetic variants have been associated with circulating 25(OH)D concentrations in cross-sectional studies, only few studies investigated possible associations between these variants and the response to vitamin D supplementation.

In some cases the studies focussed on either young individuals (19–22), other patient groups (23), or involved small number of individuals (24). A meta-analysis of 16 randomised placebo-controlled trials involving 2,994 individuals from a variety of populations (Asian, European, Brazilian, and non-Hispanic White Americans) investigated the association between FokI, BsmI, ApaI, and TaqI variants on *GC* gene and the response to vitamin D supplementation. None of the gene variant studies were significantly associated with circulating 25(OH)D concentration post-supplementation. However, several significant associations were found in subgroups when stratified by age, sex, BMI, or health status (25). In a study with 245 Tunisian participants (26), no association between *GC* variants, including rs4588, and the response to vitamin D supplementation was found. Associations between genetic variants in *CYP2R1* and *CYP24R1* and the response to vitamin D supplementation were investigated in various studies including more than 12,000 individuals from different populations (Caucasian, Asian, Arabs). The results were either inconclusive or showed that the presence of genetic variants in these genes were associated with lower 25(OH)D concentrations compared to homozygous for the common allele after 1 year of supplementation [reviewed in Bosch et al. (27)].

To better understand the influence of genetic factors on vitamin D status and the response to supplementation, we investigated whether common genetic variants at the *GC*, *DHCR7*, and *CYP2R1* loci are associated with baseline 25(OH)D and the response in plasma 25(OH)D after vitamin D supplementation in a dose-ranging randomised controlled trial of vitamin D supplementation in older people (28).

## Study participants and methods

The study cohort comprised 323 individuals from The Vitamin D supplementation in Older People (VDOP) clinical trial (ISRCTN35648481) with genetic data available. The characteristics of the trial have been described previously (28). In summary, VDOP was a randomised, double-blind trial, in which generally healthy, community dwelling men and women over 70 years old received different dosages of vitamin D (12,000, 24,000, and 48,000 IU), once monthly for 12 months at the start of the month, which is equivalent to 400, 800, and 1,600 international units (IU) daily. To ensure compliance, participants were contacted by the study staff to remind them to take the supplements. Individuals were excluded from the study if they had previously been treated with anti-resorptive or anabolic drugs for osteoporosis in the previous 3 years; currently supplemented with calcium (>500 mg/day) or vitamin D (>400 IU/day); had experienced a fragility fracture in the previous 6 months; had a history of renal stones; bilateral hip replacement; primary hyperparathyroidism; hypercalcaemia (albumin-adjusted plasma calcium >2.60 mmol/L); hypocalcaemia (albumin-adjusted plasma calcium <2.15 mmol/L); or an estimated glomerular filtration rate <30 mL · min^−1^ · 1.73 m^−2^, as detailed by Aspray et al. (28). All participants gave written informed consent to take part in the study. The clinical trial was approved by the Tyne and Wear South Research Ethics Committee (REC, 12/NE/0050), and the study complied with the Declaration of Helsinki.

### Participant characteristics and biochemical measures

Measurements and data were collected at the time of enrolling in the trial, including age, gender, dietary calcium intake, and use of vitamin D and calcium supplements. Blood plasma samples were collected at baseline and after the 12 months of vitamin D administration using either EDTA- or lithium heparin-containing tubes. Samples at 12 months were collected at the end of the month +/− one week. Concentrations of total 25(OH)D were measured at both time points using liquid chromatography mass spectrometry; parathyroid hormone (PTH) was assessed by immunoassay (Immulite, Siemens Healthcare Diagnostics Ltd., Camberley, United Kingdom); and circulating calcium concentrations using clinical biochemistry methodology at the Newcastle upon Tyne hospitals NHS Foundation Trust (NUTH) laboratories. Detailed description of the methodology was described elsewhere (29). Assessment of vitamin D status at baseline and after 12-month supplementation took place between January and April.

### Genotyping

Genomic DNA was isolated from blood using standard protocols (30). We genotyped the SNPs rs7041 and rs2282679 (proxy SNP for rs4588) at the *GC* locus (RefSeq NM_ 000583), rs12785878 at the *DHCR7* locus (RefSeq NM_ 001360), and rs10741657 at the *CYP2R1* locus (RefSeq NM_ 024514) based on results from the SUNLIGHT Consortium. The regions flanking these SNP were amplified by PCR and sequenced at the Institute of Genetics and Cancer using an Applied Biosystems 3130XL sequencer according to standard techniques (31). Primers for PCR and sequencing were designed using Primer3 v4.0.0 software (32, 33). These are shown in Supplementary Table S1. Dependence between loci were assessed by Pearson correlation (Supplementary Table S2).

### Statistical analysis

Statistical analysis was carried out using R version 4.5.0. Hardy–Weinberg equilibrium was calculated by Chi square test using the online calculator https://ug.sebc.me/labs/hwe-calculator. The statistical analysis of baseline clinical characteristics was performed using one-way ANOVA for continuous variables and Pearson’s Chi-square test for categorical variables. Tukey’s *post hoc* analysis was performed for statistically significant overall differences.

Based on the UK scientific advisory committee of nutrition (SACN) (6), categories of 25(OH)D were as <25 nmol/L (deficient); 25 to 50 nmol/L (insufficient), and >50 nmol/L (sufficient).

The relationship between genotype and baseline plasma 25(OH)D was analysed by one-way ANOVA.

To assess the relation between genotype and response to vitamin D supplementation we used linear regression analysis with post-treatment 25(OH)D as the dependent variable, genotype as independent variable of interest, and baseline 25(OH)D in interaction with dose of supplement as adjusting covariates. For the binary outcome, analysis was stratified for treatment group and a Cochrane-Mantel–Haenszel was used for the overall test. For the three main outcomes (association at baseline, after supplementation, and category of 25(OH)D concentration as based on below or above 50 nmol/L), omnibus *p*-values were adjusted for multiple comparisons using the Benjamini-Hochberg false discovery rate (FDR) correction. We considered *p*-values below 0.05 as significant. Sensitivity analyses with respect to dependence between loci were conducted by modelling the influence of multiple loci in the same model, and with respect to patient heterogeneity at baseline and follow-up by additionally adjusting for age, sex, body mass index (BMI), and dietary vitamin D intake.

## Results

### Characteristics of the cohort

The baseline characteristics of the study participants including age, sex, alcohol intake, smoking, BMI, vitamin D, PTH, circulating calcium concentrations, dietary calcium intake, vitamin D intake 25(OH)D concentrations and genotypes at the four SNPs of interest are shown in Table 1. They were recruited from a single centre in the northwest of England and were predominantly (>95%) of White background. There were no differences in these characteristics between the treatment groups or in distribution of genotypes for the four SNP tested, apart from BMI, which was higher in the 24,000 and 48,000 IU supplementation groups compared with the 12,000 IU group (*p* = 0.02). All genotypes were in Hardy–Weinberg equilibrium (Supplementary Table S3). At baseline, a total of 89/322 (27.6%) individuals had 25(OH)D concentrations below 25 nmol/L; 144/322 (44.7%) had 25–50 nmol/L concentrations, and 89 (27.6%) had 25(OH)D concentrations above 50 nmol/L. These were distributed equally between the treatment groups. Information on the vitamin D concentration at baseline was missing from one individual.

**Table 1:** Baseline characteristics of the study participants by supplementation group.

Vitamin D dose	*n*	12,000 IU/month	*n*	24,000 IU/month	*n*	48,000 IU/month	*p* value
Age (years)	104	74.6 ± 4.0	109	75.1 ± 4.3	110	75.8 ± 4.6	0.14
Sex (female)	104	50 (48.1%)	109	54 (49.5%)	110	55 (50.0%)	0.96
Alcohol (units/week)	81	7.0 [3.0–11.0]	87	7.0 [3.0–18.0]	89	10.0 [4.0–15.0]	0.22
Smoke (yes)	104	45 (43.3%)	109	54 (49.5%)	110	55 (50.0%)	0.55
BMI (kg/m^2^)	104	26.2 ± 3.6	109	27.7 ± 4.3	110	27.2 ± 4.0	0.02
Plasma 25(OH)D (nmol/L)	104	41.8 ± 20.6	108	39.3 ± 21.1	110	38.4 ± 19.6	0.47
Plasma 25(OH)D < 25 nmol/L (%)	104	28 (26.9%)	108	30 (27.8%)	110	31 (28.2%)	0.66
Plasma 25(OH)D 25–50 nmol/L (%)		42 (40.4%)		49 (45.4%)		53 (48.2%)	
Plasma 25(OH)D > 50 nmol/L (%)		34 (32.7%)		29 (26.8%)		26 (23.6%)	
PTH (pg/mL)	104	46.7 ± 24.0	107	47.9 ± 24.1	110	50.9 ± 22.0	0.37
Calcium (mmol/L)	104	2.24 ± 0.08	109	2.23 ± 0.07	109	2.24 ± 0.07	0.55
Dietary calcium (mg/day)	101	821 ± 362	107	820 ± 400	108	843 ± 347	0.88
Dietary vitamin D (IU)	101	143.5 ± 75.4	107	148.6 ± 96.0	108	157.6 ± 120.6	0.58
rs2282679 (AA/AC/CC) (*n*) in *GC*	104	51/47/6	109	65/37/7	110	55/48/7	0.49
rs7041 (CC/CA/AA) (*n*) in *GC*	104	27/58/19	109	36/56/17	110	34/54/22	0.74
rs12785878 (TT/TG/GG) (*n*) in *DHCR7*	104	61/36/7	109	61/43/5	110	72/33/5	0.57
rs10741657 (GG/GA/AA) (*n*) in *CYP2R1*	104	39/41/24	109	32/57/20	110	45/48/17	0.21

*Values are mean ± SD or numbers (%). The number of observations for each variable at each dose category are indicated. Differences in continuous variables were tested by ANOVA whilst differences in categorical variables were tested by Chi-squared test. Values for genotypes for the individual SNP are numbers of individuals with genotype for the most common allele shown first.

### Association between genotypes and baseline vitamin D concentrations

Plasma concentrations of 25(OH)D at baseline by genotype are shown in Table 2 and Supplementary Table S4. There was no significant difference in baseline 25(OH)D concentrations with the SNP rs2282679 (proxy for rs4588) in *GC*, rs12785878 in *DHCR7*, or rs10741657 in *CYP2R1*. For rs7041 in *GC*, plasma 25(OH)D concentrations differed between genotypes (*p* = 0.01 after FDR correction) with lowest values in homozygotes for the rare A allele when compared to 25(OH)D concentrations in individuals with the CC genotype (*β* (se, 95%CI) = 11.18 (3.36, 328–19.09), *p* < 0.01). Similar results were obtained when using alternative genetic models (Supplementary Table S5). rs2282679 and rs12785878 were not tested for these models due to their low number of rare homozygotes.

**Table 2:** Relation between *GC*, *DHRC7* and *CYP2R1* genotype and baseline plasma 25(OH)D concentrations.

SNP	Locus	Location	Alleles	Common homozygotes	*n*	Heterozygotes	*n*	Rare homozygotes	*n*	*p*-value	FDR *p*
rs2282679	*GC*	Intronic variant	A/C	41.3 ± 21.4	171	38.7 ± 20.0	131	34.1 ± 12.2	20	0.23	0.39
rs7041	*GC*	Exon p.(Asp432Glu)	C/A	44.8 ± 23.1	97	39.0 ± 19.5	168	33.7 ± 15.8**	57	0.003	0.01
rs12785878	*DHRC7*	Intronic variant	T/G	40.0 ± 20.5	194	39.0 ± 20.5	111	42.3 ± 20.2	17	0.81	0.88
rs10741657	*CYP2R1*	Upstream variant	G/A	38.1 ± 18.4	116	40.8 ± 19.9	145	40.4 ± 24.9	61	0.55	0.73

25(OH)D concentrations are shown as nmol/L. FDR *p* shows the *p* value after Benjamini-Hochberg adjustment. A saturated model (2-degree-of-freedom genotypic test) was used. For rs2282679 the genotypes are AA/AC/CC; for rs7041 they are CC/CA/AA; for rs12785878 they are TT/TG/GG; and for rs10741657 they are GG/GA/AA. **p < 0.01 compared to common homozygotes group (mean difference −11.2; 95% CI −19.1, −3.3). Differences between genotypes were tested by ANOVA. Data shown as mean ± standard deviation.

### Effects of vitamin D supplementation on circulating 25(OH)D concentrations according to genotype

Plasma 25(OH)D concentrations post-supplementation according to genotype and supplementation dose are shown in Table 3 and Supplementary Table S4. Overall, the mean ± SD 25(OH)D concentrations after supplementation were 56.1 ± 16.0 nmol/L with 12,000 IU, 64.2 ± 15.5 nmol/L with 24,000 IU, and 78.9 ± 14.6 nmol/L with 48,000 IU (*p* < 0.001). Adjusting for the baseline concentrations, the concentration of 25(OH)D at 12 months exhibited highly significant differences between doses (*p* < 0.001), with no significant interaction detected between dose and genotype at any of the genetic variants. The *p*-values for interaction were 0.627 for rs2282679, 0.119 for rs7041, 0.173 for rs12785878 and 0.090 for rs10741657.

**Table 3:** Plasma concentrations of 25(OH)D after supplementation according to dose and genotype.

SNP	Locus	Common homozygotes		Heterozygotes		Rare homozygotes	
		*N*	Mean ± SD	*N*	Mean ± SD	*N*	Mean ± SD
12,000 IU							
rs2282679	*GC*	51	59.7 ± 17.9	46	53.5 ± 13.6	6	44.5 ± 7.0
rs7041	*GC*	27	62.0 ± 16.8	57	56.2 ± 16.2	19	47.3 ± 9.8
rs12785878	*DHRC7*	60	58.0 ± 16.8	36	52.6 ± 15.0	7	57.0 ± 13.3
rs10741657	*CYP2R1*	39	57.5 ± 15.2	40	56.1 ± 14.8	24	53.7 ± 19.5
24,000 IU							
rs2282679	*GC*	65	68.3 ± 14.7	37	58.2 ± 14.9	7	58.5 ± 14.6
rs7041	*GC*	36	68.1 ± 12.6	56	64.0 ± 17.0	17	56.8 ± 13.2
rs12785878	*DHRC7*	61	63.0 ± 15.6	43	67.0 ± 14.6	5	54.9 ± 19.0
rs10741657	*CYP2R1*	32	59.0 ± 14.2	57	67.0 ± 14.1	20	64.7 ± 19.4
48,000 IU							
rs2282679	*GC*	52	81.8 ± 16.7	48	76.2 ± 12.1	7	76.6 ± 10.6
rs7041	*GC*	32	82.4 ± 15.8	53	78.1 ± 15.2	22	75.7 ± 10.5
rs12785878	*DHRC7*	70	78.5 ± 15.4	32	79.6 ± 13.4	5	80.1 ± 13.2
rs10741657	*CYP2R1*	45	74.4 ± 13.3	45	81.4 ± 15.9	17	84.4 ± 11.4

Values are mean ± standard deviation. A saturated model (2-degree-of-freedom genotypic test) was used. There was a dose-dependent increase in 25(OH)D concentration with the vitamin D supplements (*p* < 0.001). Of the variants analysed, alleles of rs7041 and rs2282679 were significantly associated with 25(OH)D concentrations after supplementation overall (*p* < 0.001 and *p* = 0.02 after Benjamini-Hochberg adjustment, respectively). Tukey’s post hoc analysis showed significant differences between common homozygote and both heterozygote (least square mean difference −6.6; 95% CI −9.9, −3.2; *p* < 0.001), and rare homozygote (least square mean difference −6.8; 95% CI −13.5, −0.04; *p* = 0.048) for rs2282679, as well as between the common and the rare heterozygotes (least square mean difference −6.7; 95% CI −11.7, −1.8; *p* = 0.004) for rs7041. There was no significant difference for alleles at variants rs12785878 (unadjusted *p* = 0.50) or rs10741657 (unadjusted *p* = 0.06).

**Figure 1:**
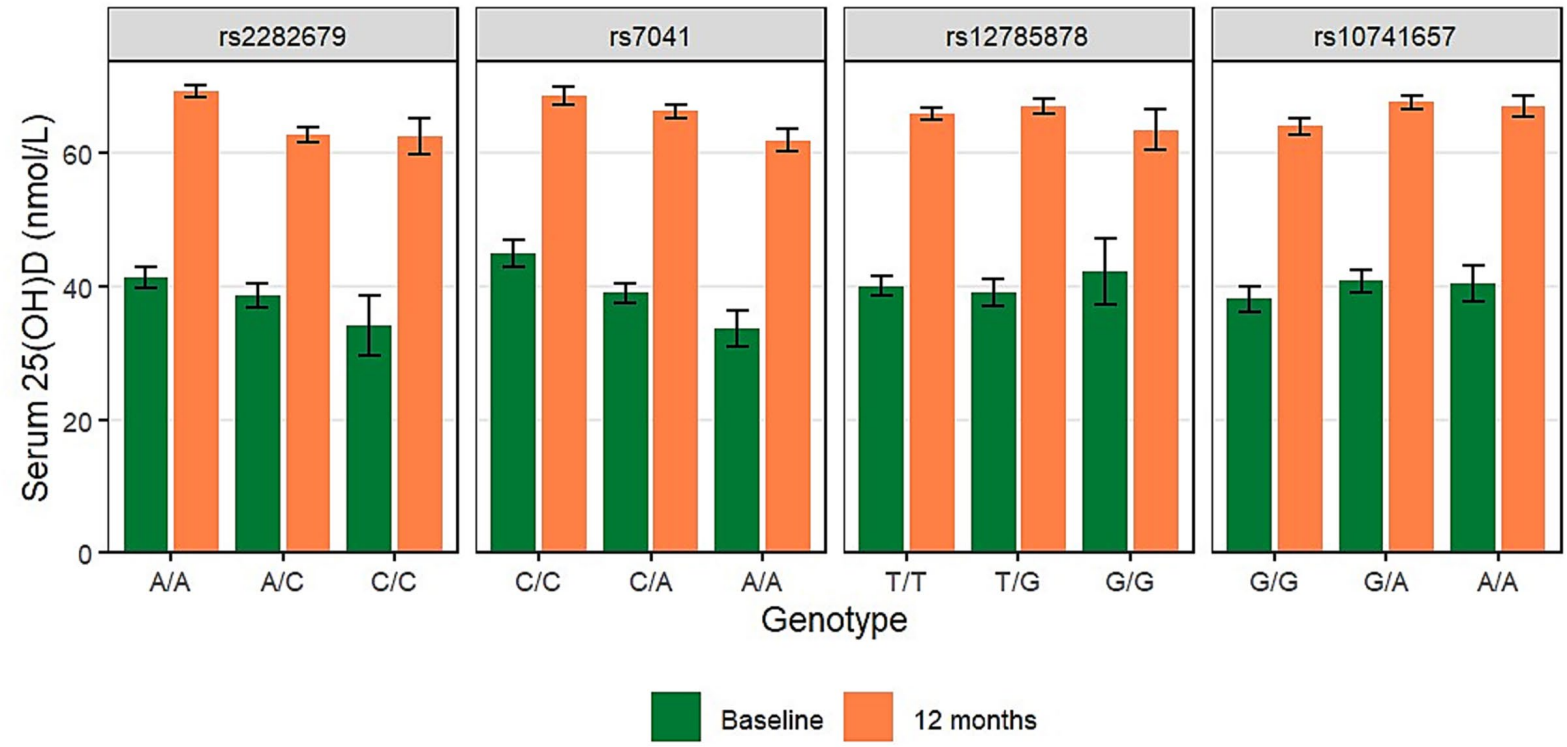
Plasma 25(OH) concentrations before and after supplementation in relation to variants at the *GC, CYP2R1,* and *DHCR7* loci. Columns are least square means and standard errors of the mean of plasma 25(OH)D concentrations per genotype for each of the analysed variants at both, baseline and after supplementation. Least square means for the 12-month timepoint were calculated adjusting for supplementation group, baseline vitamin D, and their interaction.

We observed a significant difference in plasma 25(OH)D concentration following supplementation at all dose allocations according to alleles at rs2282679 in the *GC* locus. Individuals carrying two copies of the common allele presented higher concentrations of 25(OH)D with evidence of an allele dose effect (Table 3; Supplementary Table S4; Figure 1). Similarly, plasma 25(OH)D concentrations following supplementation were higher in those who carried the common allele of rs7041 with evidence of an allele dose effect, especially when compared to individuals homozygous for the rare allele (Table 3; Supplementary Table S4; Figure 1). In contrast, there was no significant difference in 25(OH)D concentration according to alleles at the *DHRC7* variant rs12785878 or the *CYP2R1* variant rs10741657 (Table 3; Supplementary Table S4; Figure 1). Similar results were obtained when tested by alternative genetic models. Although rs10741657 was found significantly associated with changes in 25(OH)D after supplementation using a dominant model (AIC = 2508.05 vs. 2509.92 in the saturated model), this difference was not significant (*p* = 0.73), as shown in Supplementary Table S6.

The results of the analyses of the relation between genotype and 25(OH)D category after supplementation is summarised in Table 4. Only 1 individual had a 25(OH)D below 25 nmol/L following vitamin D supplementation. Therefore, we combined data for participants with concentrations below 25 nmol/L and between 25 and 50 nmoL/L. In the group 48,000 IU, all participants had a 25(OH)D above 50 nmol/L following supplementation. Homozygotes for the minor allele for variants rs2282679 and rs7041 at *GC* gene were associated with concentrations below 50 nmol/L. Stratified analysis per group of vitamin D supplementation (Supplementary Table S7) showed that the minor allele for SNPs rs2282679 (proxy for rs4588) was significantly associated with 25(OH)D concentrations below 50 nmol/L after vitamin D supplementation in the groups 12,000 IU and 24,000 IU, while the minor allele for SNPs rs7041 in *GC* locus exhibited significant association only at 12,000 IU. No significant associations were found for the tested SNPs in *DHRC7* and *CYP2R1* loci.

**Table 4:** Relation between genotype and category of plasma 25(OH)D post-supplementation.

SNP	Locus	*N* (*n* missing)	25(OH)D < 50 nmol/L	25(OH)D > 50 nmol/L	OR (95% CI)	*p*-value	FDR p
rs2282679 (AA)	*GC*	171 (3)	20 (11.7%)	148 (86.5%)	2.97 (1.56–5.65)	0.001	0.007
rs2282679 (AC/CC)		152 (1)	38 (25%)	113 (74.3%)			
rs7041 (CC)	*GC*	97 (2)	9 (9.3%)	86 (88.7%)	2.84 (1.28–6.32)	0.014	0.03
rs7041 (CA/AA)		226 (2)	49 (21.7%)	175 (77.4%)			
rs12785878 (TT)	*DHRC7*	194 (3)	34 (17.5%)	157 (80.9%)	0.93 (0.50–1.74)	0.96	0.96
rs12785878 (TG/GG)		129 (1)	24 (18.6%)	104 (80.6%)			
rs10741657 (GG)	*CYP2R1*	116	22 (19%)	94 (81%)	0.84 (0.45–1.56)	0.68	0.82
rs10741657 (GA/AA)		207 (4)	36 (17.4%)	167 (80.7%)			

Values are shown as *n* (%) excluding missing values. FDR *p* shows the *p* value after Benjamini-Hochberg adjustment. Due to the low sample size of the rare homozygotes, they were merged with heterozygotes for statistical analysis, which was performed using the Cochran–Mantel–Haenszel test, comparing the common vs. the merged heterozygous and rare allele groups and stratifying by treatment group.

### Sensitivity analyses

Due to the significant dependence between loci rs2282679 and rs7041 (Pearson correlation *R*^2^ = 0.47; *p* < 0.001), we investigated the robustness of the main effect with respect to inclusion of these two SNPs in the same model. This sensitivity analysis corroborated the results of the main analysis for the baseline vitamin D plasma concentrations, with *p* = 0.65 and *p* = 0.009 for rs2282679 and rs7041, respectively. For the 12-month timepoint, only rs2282679 remained significant (*p* < 0.001), whilst rs7041 was no longer associated (*p* = 0.25) (Supplementary Table S8). Sensitivity analyses with respect to the adjustment for age, gender, BMI, and dietary vitamin D intake did not qualitatively change the results from the main analysis (Supplementary Table S9).

## Discussion

In this study we evaluated the relation between common genetic variants at three loci that have been associated with circulating 25(OH)D concentrations at a genome wide significant level in previous studies as determinants of response to vitamin D supplementation. The study used data and samples from the VDOP study, where older, healthy individuals were supplemented with monthly doses of 12,000 IU, 24,000 IU and 48,000 IU colecalciferol for 1 year.

As previously reported (28, 29), the circulating concentrations of 25(OH)D increased after supplementation in a dose-dependent manner. The genetic analysis showed no significant association between variants at the *CYP2R1* or *DHCR7* loci and 25(OH)D at baseline or following supplementation. Variants in the rs7041 at the *GC* locus were associated with baseline 25(OH)D concentrations and following supplementation, we observed significant associations between variants rs2282679 and rs7041 at *GC* locus and plasma 25(OH)D concentrations, a link mostly driven by the former variant. For rs2282679 (proxy for rs4588), 25(OH)D concentrations were highest in common homozygotes with an allele dose response. Similar findings were found for rs7041. In keeping with this, homozygotes for the common allele of rs2282679 were significantly less likely to have 25(OH)D concentrations < 50 nmol/L after supplementation compared to heterozygotes and rare homozygotes (11.7% vs. 25%) and the same was true for homozygotes for the common allele at rs7041 (9.3% vs. 21.7%). No association between the *DHRC7* variant rs12785878 or the *CYP2R1* variant rs10741657 with 25(OH)D concentration was observed following supplementation.

Previous studies have identified associations between 25(OH)D and both rs7041 and rs2282679 in the *GC* locus in European White populations (8, 34, 35), and in smaller studies involving African and American Black and Asian populations (36, 37). Such link could be influenced by the considerable differences in the distribution of *GC* isotypes (as composed of the variants Gc1f, Gc1S and Gc2 (38), giving rise to six combinations). Distribution of these isotypes vary amongst populations of different racial background. Black Americans and Black-Gambians have a 1F allele whilst White people predominantly present 1S1S and 1S2 genotypes but not 1F (13). In a previous study reported by Carpenter et al. (39) the common allele at rs4588 variant (and by implication the common allele at the rs2282679 variant) was associated with higher serum concentrations of VDBP and 25(OH)D in an observational study in paediatric population as compared with the rare allele. The same study reported that variants at rs7041 were not associated with serum VDBP but were associated with total 25(OH)D. These differences could be linked to the genotype-driven variations in VDBP concentration or 25(OH)D half-life, as seen in both observational (13, 38) and interventional studies in different populations (40, 41).

The substitution of lysine for threonine at position 436 eliminates an O-glycosylation site from the molecule and there is evidence that loss of glycosylation may affect VDBP stability and binding of vitamin D, leading to changes in the concentration and internalisation and thus bioavailability of circulating 25(OH)D (42).

It was of interest that we did not observe associations with SNPs close to the *CYP2R1* and *DHCR7* genes which have previously been associated with serum 25(OH)D concentration by GWAS (8). Although the cohorts included in this GWAS were all observational, the large sample size (>33,000 individuals) would have a higher power to detect smaller effect of variants. The *CYP2R1* gene encodes a hepatic enzyme responsible for the 25-hydroxylation of vitamin D in the liver and the lack of association between variants at this locus and 25(OH)D after supplementation might possibly be explained by the fact that variations at *CYP2R1* might have less influence on 25-hydroxylation in those who have been supplemented with vitamin D. Expression of *CYP2R1* is not induced by vitamin D supplementation (43), except for exceptionally high intake concentration exceeding the hepatic hydroxylation capacity. This is thought to be caused by alterations in the methylation sites of the *CYP2R1* gene (44). The lack of association of variants close to the *DHCR7* gene, which regulates the conversion of 7-DHC into cholesterol, reducing its availability as substrate to generate sunlight-driven colecalciferol, and subsequent 25(OH)D, could be explained by the fact that serum concentration of 25(OH)D are less dependent on the cutaneous conversion of 7-DHC into pre-vitamin D when oral supplementation is taken. Assessment of vitamin D status at baseline and after 12-month supplementation took place between January and April, when the effect of sunlight on vitamin D status is much smaller than in the summer.

This study has the limitation that the influence of genetic background on the change in 25(OH)D concentration after supplementation cannot be accounted for, separately from its influence on baseline 25(OH)D concentration, which is known to play a role in the response to supplementation. Low baseline concentrations, especially below 40 nmol/L are documented to be associated with larger changes after supplementation. Considering this, we corrected all statistical analysis for baseline 25(OH)D concentration. Another limitation is the ethnic composition of the study population, including predominantly White participants. Considering the ethnic differences in the distribution of *GC* gene variants as discussed above, associations might differ in other population groups.

In summary, our study suggests that common variants at the *GC* locus could be significantly associated with 25(OH)D concentration in plasma after supplementation. In this cohort, carriers of the less common alleles at rs7041 and rs2282679 (proxy for rs4588) were more than twice as likely to have 25(OH)D concentrations below 50 nmol/L compared to those homozygous for the common alleles. Considering that several rare genotypes were underrepresented in this cohort, these results require further validation in a larger cohort. In routine clinical practise it is not uncommon to encounter patients whose 25(OH)D concentrations remain below 50 nmol/L despite taking supplements. Our study suggests that one mechanism for suboptimal concentrations of 25(OH)D after supplementation could be the genetic variation at the *GC* locus. Thus, these results emphasise the importance of further research on the role of *GC* SNPs as determinants of vitamin D status and the response to vitamin D supplementation.

## Data Availability

The datasets presented in this study can be found in online repositories. The names of the repository/repositories and accession number(s) can be found at: https://zenodo.org/, 10.5281/zenodo.20525339.
