# Supplementary material for: Genetic determinants of response to vitamin D supplementation
Source: Front Nutr. 2026 Jul 15;13:1880174. doi: 10.3389/fnut.2026.1880174 (PMC13414278; doi:10.3389/fnut.2026.1880174)
Supplement: Supplementary file 1 [file Supplementary_file_1.docx]

**Supplementary material**

**Genetic determinants of response to vitamin D supplementation**

Nerea Alonso ^1,2^, Inez Schoenmakers^3^, Thomas Kuenzer^4^, Ariane Willems ^1^, Huilin Jin^1^, Terry J Aspray ^5,6^, Stuart H Ralston ^1*^

^1^ Institute of Genetics and Cancer, University of Edinburgh, Edinburgh, UK

^2^ Clinical Institute of Medical and Chemical Laboratory Diagnostics (CIMCL), Medical University of Graz, Graz, Austria

^3^ Department of Medicine, Norwich Medical School, Faculty of Medicine and Health Sciences, University of East Anglia, Norwich NR4 7TJ, UK

^4^ Institute for Medical Informatics, Statistics and Documentation, Medical University of Graz, Graz, Austria.

^5^ NIHR Newcastle Biomedical Research Centre, Campus for Ageing and Vitality, Newcastle upon Tyne, UK

^6^ Institute for Cellular Medicine, Newcastle University, Newcastle upon Tyne, UK

**Suppl. Table S1**. Primer sequences used to amplify and sequence the genomic regions containing the variants in *GC, DHCR7*, and *CYP2R1* genes analysed.

|  | **Gene** | **Primer forward** | **Primer reverse** |
| --- | --- | --- | --- |
| **rs2282679** | *GC* | GGAGCTGGGACTACAGTTGC | CACGAATCTGTTCCCTCACA |
| **rs7041** | *GC* | TCTCGAAGAGGCATGTTTCA | TGAGTAGATTGGAGTGCATACG |
| **rs12785878** | *DHCR7* | TGTGAGTCCAGAGGCATCTTT | GACTGGGCTGTCACCACTG |
| **rs10741657** | *CYP2R1* | TGTTCCCATGTCCTAAGCAA | CTGTCAGCCCTGGAAGACTC |

**Suppl. Table S2.** Dependence between the investigated variants in *GC, DHCR7*, and *CYP2R1* genes

| **Variant 1** | **Gene** | **Variant 2** | **Gene** | **N** | **Pearson correlation** | **R^2^** | **p-value** | **Adjusted p-value** |
| --- | --- | --- | --- | --- | --- | --- | --- | --- |
| **rs2282679** | *GC* | **rs7041** | *GC* | 323 | 0.682 | 0.465 | <0.001 | <0.001 |
| **rs2282679** | *GC* | **rs12785878** | *DHCR7* | 323 | -0.041 | 0.002 | 0.47 | 0.56 |
| **rs2282679** | *GC* | **rs10741657** | *CYP2R1* | 323 | 0.079 | 0.006 | 0.15 | 0.31 |
| **rs7041** | *GC* | **rs12785878** | *DHCR7* | 323 | -0.026 | 0.001 | 0.64 | 0.64 |
| **rs7041** | *GC* | **rs10741657** | *CYP2R1* | 323 | 0.046 | 0.002 | 0.41 | 0.56 |
| **rs12785878** | *DHCR7* | **rs10741657** | *CYP2R1* | 323 | 0.093 | 0.009 | 0.10 | 0.29 |

P value was adjusted by Benjamini-Hochberg

**Suppl. Table S3.** Hardy-Weinberg equilibrium test for the analysed variants in *GC, DHCR7*, and *CYP2R1* genes

|  | **Gene** | **Common homozygotes (N)** | **Heterozygous (N)** | **Rare homozygotes (N)** | **Df** | **Chi-sq value** | **P value** |
| --- | --- | --- | --- | --- | --- | --- | --- |
| **rs2282679** | *GC* | 171 | 132 | 20 | 2 | 0.68 | 0.41 |
| **rs7041** | *GC* | 97 | 168 | 58 | 2 | 1.00 | 0.32 |
| **rs12785878** | *DHCR7* | 194 | 112 | 17 | 2 | 0.02 | 0.87 |
| **rs10741657** | *CYP2R1* | 116 | 146 | 61 | 2 | 1.54 | 0.21 |

N: number, Df: degrees-of-freedom. Chi squared test was calculated using the online Hardy-Weinberg calculator https://ug.sebc.me/labs/hwe-calculator

**Suppl. Table S4.** Pairwise comparisons between the different genotypes and the 25(OH)D concentration at both baseline and post-supplementation

|  |  | **Baseline 25(OH)D** | | **Post-supplementation 25(OH)D** | |
| --- | --- | --- | --- | --- | --- |
| **SNP** | **Genotype contrast** | **β** | **95% CI** | **β** | **95% CI** |
| **rs2282679** | **AA - AC** | 2.69 | -2.88, 8.27 | 6.57 | 3.23, 9.91 |
|  | **AA - CC** | 7.25 | -4.10, 18.59 | 6.76 | 0.04, 13.49 |
|  | **AC - CC** | 4.56 | -6.97, 16.08 | 0.19 | -6.64, 7.03 |
| **rs7041** | **AA - AC** | 5.89 | -0.15, 11.93 | 2.37 | -1.38, 6.13 |
|  | **AA - CC** | 11.18 | 3.28, 19.09 | 6.74 | 1.79, 11.68 |
|  | **AC - CC** | 5.30 | -1.96, 12.56 | 4.36 | -0.12, 8.84 |
| **rs12785878** | **TT - TG** | 0.97 | -4.76, 6.71 | -1.05 | -4.57, 2.47 |
|  | **TT - GG** | -2.25 | -14.44, 9.94 | 2.51 | -4.90, 9.93 |
|  | **TG - GG** | -3.22 | -15.77, 9.33 | 3.57 | -4.07, 11.20 |
| **rs10741657** | **GG - GA** | -2.708 | -8.70, 3.29 | -3.68 | -7.36, 0.01 |
|  | **GG - AA** | -2.279 | -9.89, 5.33 | -3.02 | -7.64, 1.60 |
|  | **GA - AA** | 0.429 | -6.92, 7.78 | 0.65 | -3.81, 5.12 |

Se: standard error, CI: confidence interval. Pairwise comparisons using Tukey method.

**Suppl. Table S5.** Model comparison for the association between genetic variants and 25(OH)D concentration at baseline.

|  | **rs7041** | | | **rs10741657** | | |
| --- | --- | --- | --- | --- | --- | --- |
| **Model** | **AIC** | **p-value** | **p-value (saturated model)** | **AID** | **p-value** | **p-value (saturated model)** |
| **Saturated** | 2851.79 | 0.003 |  | 2862.18 | 0.55 |  |
| **Additive** | 2849.81 | <0.001 | 0.90 | 2860.63 | 0.39 | 0.50 |
| **Dominant** | 2852.76 | 0.003 | 0.087 | 2860.20 | 0.28 | 0.89 |
| **Recessive** | 2855.06 | 0.012 | 0.022 | 2861.32 | 0.79 | 0.29 |

Saturated model: 2-degree-of-freedom, genotypic test. AIC: Akaike Information Criterion. Allele coding was common homozygotes, heterozygotes, rare homozygotes for the saturated and additive models, and binary for the dominant and recessive models.

**Suppl. Table S6.** Model comparison for the association between genetic variants and 25(OH)D concentration after 12-month supplementation.

|  | **rs7041** | | | **rs10741657** | | |
| --- | --- | --- | --- | --- | --- | --- |
| **Model** | **AIC** | **p-value** | **p-value (saturated model)** | **AID** | **p-value** | **p-value (saturated model)** |
| **Saturated** | 2505.47 | 0.006 |  | 2509.92 | 0.055 |  |
| **Additive** | 2503.98 | 0.002 | 0.480 | 2510.25 | 0.060 | 0.130 |
| **Dominant** | 2508.82 | 0.026 | 0.022 | 2508.05 | 0.017 | 0.730 |
| **Recessive** | 2505.73 | 0.005 | 0.140 | 2513.54 | 0.580 | 0.019 |

Saturated model: 2-degree-of-freedom, genotypic test. AIC: Akaike Information Criterion. Multivariable models adjusted for baseline 25(OH)D concentration, dose group and their interaction. Allele coding was common homozygotes, heterozygotes, rare homozygotes for the saturated and additive models, and binary for the dominant and recessive models.

**Suppl. Table S7.** Relationship between 25(OH)D concentration < 50 nmol/L and genotype per vitamin D dose.

| **Dose** | **SNP** |  | **25(OH)D < 50 nmol/L, n (%)** | | |  | **Heterozygous vs common** | | **Rare vs common** | | **Rare + Heterozygous vs Common** | |
| --- | --- | --- | --- | --- | --- | --- | --- | --- | --- | --- | --- | --- |
|  |  | **Locus** | **Common homozygote** | **Heterozygote** | **Rare homozygote** | **p-value** | **Risk ratio** | **p** | **Risk ratio** | **p** | **Risk ratio** | **p** |
| **12000 IU** | **rs2282679** | *GC* | 14/51 (27.5%) | 19/46 (41.3%) | 5/6 (83.3%) | 0.019 | 1.50 (0.94-2.40) | 0.094 | 3.04 (1.88-4.89) | 0.004 | 1.68 (1.09-2.59) | 0.020 |
|  | **rs7041** | *GC* | 6/27 (22.2%) | 17/57 (29.8%) | 15/19 (78.9%) | <0.001 | 1.34 (0.71-2.54) | 0.36 | 3.55 (2.05-6.16) | <0.001 | 1.89 (1.08-3.33) | 0.018 |
|  | **rs12785878** | *DHRC7* | 20/60 (33.3%) | 15/36 (41.7%) | 3/7 (42.9%) | 0.675 | 1.25 (0.79-1.98) | 0.36 | 1.29 (0.53-3.14) | 0.60 | 1.26 (0.81-1.94) | 0.32 |
|  | **rs10741657** | *CYP2R1* | 12/39 (30.8%) | 15/40 (37.5%) | 11/24 (45.8%) | 0.482 | 1.22 (0.72-2.05) | 0.46 | 1.49 (0.86-2.58) | 0.17 | 1.32 (0.85-2.06) | 0.22 |
| **24000 IU** | **rs2282679** | *GC* | 6/65 (9.2%) | 12/37 (32.4%) | 2/7 (28.6%) | 0.011 | 3.51 (1.72-7.16) | <0.001 | 3.10 (0.85-11.24) | 0.10 | 3.45 (1.73-6.88) | <0.001 |
|  | **rs7041** | *GC* | 3/36 (8.3%) | 12/56 (21.4%) | 5/17 (29.4%) | 0.125 | 2.57 (1.03-6.42) | 0.034 | 3.53 (1.22-10.21) | 0.018 | 2.79 (1.17-6.68) | 0.014 |
|  | **rs12785878** | *DHRC7* | 14/61 (23.0%) | 4/43 (9.3%) | 2/5 (40.0%) | 0.092 | 0.41 (0.15-1.09) | 0.052 | 1.74 (0.57-5.35) | 0.38 | 0.54 (0.24-1.23) | 0.13 |
|  | **rs10741657** | *CYP2R1* | 10/32 (31.2%) | 6/57 (10.5%) | 4/20 (20.0%) | 0.052 | 0.34 (0.15-0.78) | 0.006 | 0.64 (0.25-1.65) | 0.33 | 0.42 (0.21-0.82) | 0.008 |

Supplementation group of 48,000 IU did not contain any participant showing plasma 25(OH)D concentrations < 50 nmol/L

**Suppl. Table S8.** Analysis of the association between variants in *GC* gene and 25(OH)D concentrations at baseline and post-supplementation.

|  | **Baseline 25(OH)D** | | | **Post-supplementation 25(OH)D*** | | |
| --- | --- | --- | --- | --- | --- | --- |
| **Variant** | **β** | **95%CI** | **p-value** | **β** | **95%CI** | **p-value** |
| **rs2282679** |  |  | 0.65 |  |  | <0.001 |
| Heterozygote | 2.7 | -3.1, 8.6 | 0.36 | -7.0 | -11.0, -3.5 | <0.001 |
| Rare homozygote | 2.9 | -9.1, 15 | 0.64 | -4.8 | -12.0, 2.4 | 0.19 |
| **rs7041** |  |  | 0.009 |  |  | 0.25 |
| Heterozygote | -7.5 | -14.0, -1.4 | 0.017 | 1.9 | -1.9, 5.6 | 0.32 |
| Rare homozygote | -14.0 | -23.0, -4.6 | 0.003 | -1.3 | -6.8, 4.2 | 0.64 |

*Post-supplementation model was adjusted for baseline 25(OH)D concentration, dosage group, and their interaction.

**Suppl. Table S9.** Sensitivity analysis of changes in 25(OH)D concentration at both baseline and after 12-month supplementation.

|  | **Baseline 25(OH)D** | | | **Post-supplementation 25(OH)D** | | |
| --- | --- | --- | --- | --- | --- | --- |
| **Variant** | **N** | **β (95%CI)** | **p-value** | **N** | **β (95%CI)** | **p-value** |
| **rs2282679** | 315 |  | 0.12 | 302 |  | <0.001 |
| Heterozygotes |  | -3.8 (-8.4, 0.76) | 0.10 |  | -6.7 (-9.4, -3.9) | <0.001 |
| Rare homozygotes |  | -7.3 (-17.0, 1.9) | 0.12 |  | -8.3 (-14.0, -2.7) | 0.004 |
| **rs7041** | 315 |  | 0.002 | 302 |  | 0.002 |
| Heterozygotes |  | -6.4 (-11.0, -1.5) | 0.011 |  | -2.5 (-5.6, 0.64) | 0.12 |
| Rare homozygotes |  | -11.0 (-18.0, -4.7) | <0.001 |  | -7.5 (-12.0, -3.4) | <0.001 |
| **rs12785878** | 315 |  | 0.80 | 302 |  | 0.60 |
| Heterozygotes |  | -1.5 (-6.2, 3.2) | 0.53 |  | 0.34 (-2.6, 3.3) | 0.82 |
| Rare homozygotes |  | 0.48 (-9.4, 10.0) | 0.92 |  | -2.9 (-9.1, 3.3) | 0.35 |
| **rs10741657** | 315 |  | 0.70 | 302 |  | 0.045 |
| Heterozygotes |  | 2.0 (-2.9, 6.9) | 0.43 |  | 3.8 (0.73, 6.8) | 0.015 |
| Rare homozygotes |  | 0.20 (-6.1, 6.6) | 0.95 |  | 3.1 (-0.78, 7.0) | 0.12 |

Analysis adjusted for age, sex, BMI, and dietary vitamin D intake. *Post-supplementation model was adjusted for baseline 25(OH)D concentration, dosage group, and their interaction.CI: confidence interval
